# Personal

**Published:** 1907-06

**Authors:** 


### PERSONAL.

DR. ALBERT VANDER VEER, of Albany, professor of clinical and abdominal surgery in Albany Medical College, regent of the University of the State of New York, was tendered a dinner at the Hotel Ten Eyck, Albany, May 2, 1907, by more than one hundred of his former pupils. The chief speakers were Dr. Joseph D. Bryant and Dr. Thomas H. Willard, of New York; Dr. Frederic C. Curtis, Dr. Thomas Wilson and Dr. Samuel B. Ward, of Albany. Dr. Willard was the chief organiser of the banquet.

---

DR. BENJAMIN H. GROVE, of Buffalo, will spend the months of July and August in European travel. Dr. Grove is one of the leading ophthalmologists in this city and has earned the pleasant vacation which he contemplates.

---

DR. H. Y. GRANT, of Buffalo, who has practised ophthalmology in this city for some years, is about to sail for Europe, accompanied by Mrs. Grant, and will remain abroad for a number of months. Dr. Grant is a son of Sir James A. Grant, of Ottawa, whose distinguished place in medicine has brought him the honors of knighthood.

---

DR. JOHN CHALMERS, of Buffalo, has removed his offices and residence from 321 West Ferry street to 328 West Ferry street. Hours: 8 to 9; 2 to 3, and 7 to 8. Sundays, 1 to 3. Telephone, North 3712.

---

DR. JAMES A. MACLEOD, of Buffalo, has removed his offices from 482 Delaware Avenue to the Hotel Touraine Annex, 262 Delaware Avenue. He will continue to reside at the Hotel Touraine. Hours: 1 to 3, and by appointment.

---

DR. JOHN J. TWOHEY, of Buffalo, announces the removal of his offices and residence from 2200 Main Street to 2148 Main street. Telephone, Park 121.

---

DR. F. FOLLONSBEE, of this city, has been appointed house surgeon at Riverside Hospital to serve one year from May 1, 1907.

---

DR. WILLIAM H. WATSON, of Utica, formerly a regent of the University of the state of New York, has been elected an honorary member of the Russian Surgical Society, which celebrated its twenty-fifth anniversary May 9, 1907.

---

DR. NICHOLAS SENN, of Chicago, was elected to honorary fellowship in the Russian Surgical Society, which recently celebrated its twenty-fifth anniversary at St. Petersburg.

---

DR. WILLIAM HOUSE, formerly of Buffalo, announces to the physicians of the Pacific Northwest that, after three and one-half years spent as resident physician at Crystal Springs Sanitarium, Portland, Oregon, he has retired from that institution and has taken offices at 313-314 Oregonian Building, Portland, Oregon. Dr. House will devote special attention to diseases of the brain and nervous system to which he has given seven years exclusively as resident physician in various institutions.

---

DR. E. C. DUDLEY of Chicago, has removed his offices to room 116, Reliance building, southwest corner of State and Washington streets. Consultation hours, twelve to one and two to three P.M. House address, 1619 Indiana avenue.

